# P-1573. Healthcare Costs Associated With Lyme Disease Among Medicare Fee-for-Service Beneficiaries in the United States: A Retrospective Claims-Based Study

**DOI:** 10.1093/ofid/ofaf695.1753

**Published:** 2026-01-11

**Authors:** Holly Yu, Peter Kardel, Heidi De Souza, L Hannah Gould

**Affiliations:** Pfizer Inc., Collegeville, PA; ADVI Health LLC, Washington, District of Columbia; ADVI Health LLC, Washington, District of Columbia; Pfizer Vaccines, New York, New York

## Abstract

**Background:**

Lyme disease (LD) is the most common tick-borne illness in the United States (US). Incidence peaks in older adults, yet LD-associated healthcare costs are understudied in this population. This retrospective, observational study assessed LD-associated healthcare costs among US Medicare Fee-for-Service (FFS) beneficiaries aged ≥ 65 years.

**Figure:**
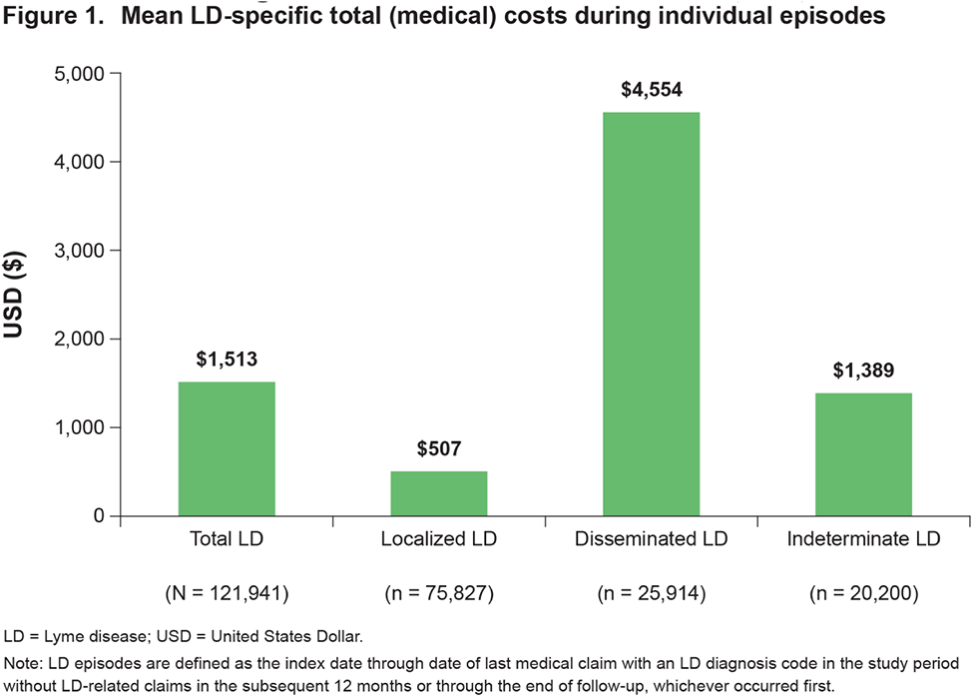

**Figure:**
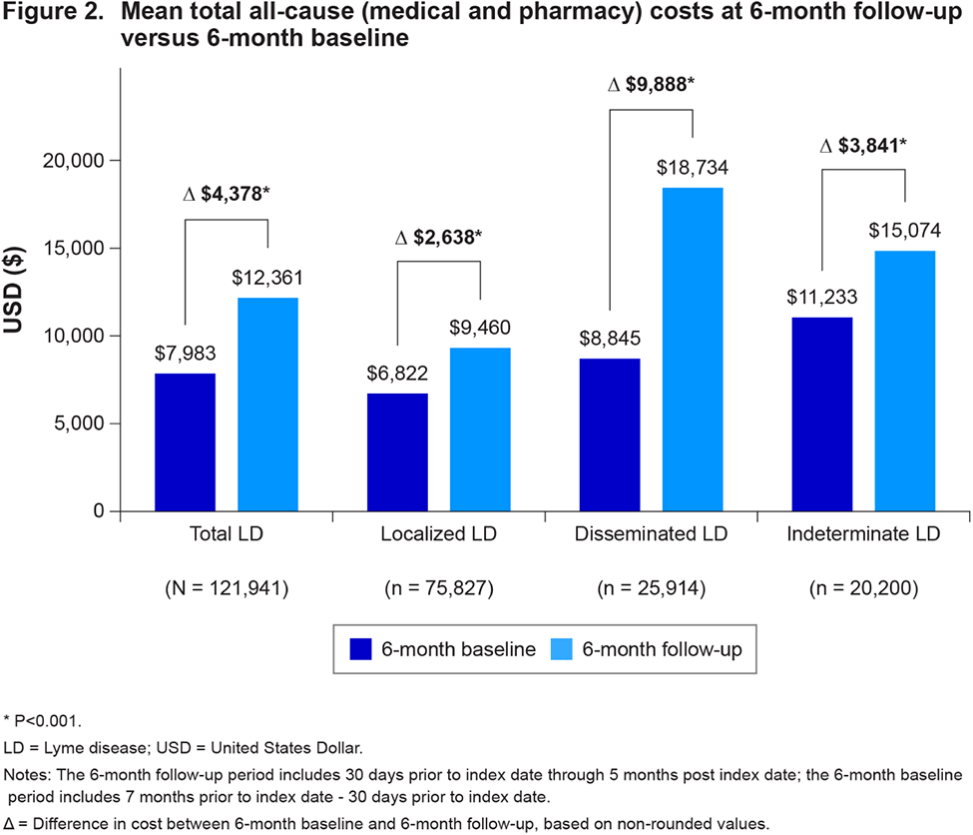

**Methods:**

Eligible LD cases were identified in Medicare FFS claims data (Medicare Parts A, B, and D) from Jan 2016–Jul 2023, had continuous eligibility for the assessment period, and had ≥ 1 outpatient or inpatient LD claim. Outpatient claims had an LD diagnosis code (A69.xx) with qualifying antibiotic treatment within 30 days. Inpatient claims had a primary LD diagnosis code or a secondary LD diagnosis code with a primary LD-linked condition. Cases were classified into subgroups (localized, disseminated, indeterminate) based on LD-associated diagnosis codes, outpatient and inpatient services, and antibiotics. Outcomes included LD-specific (medical) costs during individual LD episodes and all-cause (medical and pharmacy) costs at 6-month follow-up versus 6-month baseline. Comparisons used a paired *t* test for continuous measures, with significance noted at *P*< 0.001.

**Results:**

Among 121,941 identified LD cases, 53.0% were female, 93.4% were White, and the mean (standard deviation) age was 74.0 (6.0) years. Of these, 62.2% had localized LD, 21.3% had disseminated LD, and 16.6% had indeterminate LD. Mean LD-specific total (medical) costs were $1,513 overall and were highest for disseminated cases ($4,554) (Figure 1). Mean all-cause total costs at 6-month follow-up versus 6-month baseline were significantly higher for LD cases overall (Δ$4,378), localized LD cases (Δ$2,638), disseminated LD cases (Δ$9,888), and indeterminate LD cases (Δ$3,841) (Figure 2).

**Conclusion:**

US Medicare FFS beneficiaries with LD incurred substantial healthcare costs, especially those with disseminated disease. Strategies to prevent LD cases in older adults may decrease associated economic burden.

**Disclosures:**

Holly Yu, MSPH, Pfizer Inc.: Employee; may hold company shares and/or stocks. Peter Kardel, MA, ADVI Health LLC: Employee; may hold company shares and/or stocks. Heidi De Souza, MPH, ADVI Health LLC: Employee; may hold company shares and/or stocks. L. Hannah Gould, PhD, MS, MBA, Pfizer Inc.: Employee; may hold company shares and/or stocks.

